# Coding-complete genome sequence of a bovine viral diarrhea virus subgenotype 1v strain isolated in China

**DOI:** 10.1128/mra.01150-24

**Published:** 2025-02-03

**Authors:** Shandian Gao, Wei Liu, Junjun Shao, Yun Zhang, Huichen Guo, Huiyun Chang, Jiandong Wang

**Affiliations:** 1 Gansu Province Research Center for Basic Disciplines of Pathogen Biology, State Key Laboratory for Animal Disease Control and Prevention, Lanzhou Veterinary Research Institute, Chinese Academy of Agricultural Sciences, Lanzhou, Gansu, China; 2 Institute of Animal Science, Ningxia Academy of Agricultural and Forestry Sciences, Yinchuan, China; Katholieke Universiteit Leuven, Leuven, Belgium

**Keywords:** bovine viral diarrhea virus, coding-complete genome sequence, subgenotype

## Abstract

We sequenced the coding-complete genome sequence of bovine viral diarrhea virus (BVDV) strain NX2019/02. It belongs to the 1v subgenotype. This report will increase our understanding of the epidemiology of BVDV in China.

## ANNOUNCEMENT

Bovine viral diarrhea virus (BVDV) is one of the most widely distributed pathogenic viruses throughout the world (1). It belongs to the *Flaviviridae* family, *Pestivirus* genus. Based on the antigenic and genetic differences, the viruses are classified into several genotypes, including BVDV-1 (*Pestivirus* A), BVDV-2 (*Pestivirus* B), and BVDV-3 (*Pestivirus* H) based on nucleotide or amino acid sequence distances of the complete coding sequences, antigenic differences, and natural host range (2, 3). The BVDV-1 genotype is dominant worldwide and over 23 subgenotypes from 1a to 1w have been reported (4–6). To date, the latest BVDV-1v subgenotype was identified only in China. It was first reported in Heilongjiang Province in the northwest (7) and now has been reported in different provinces including Shandong, Inner Mongolia (6), Sichuan, Henan, Beijing (8), Hebei (9), and the Ningxia Hui Autonomous Region (10), highlighting the need for surveillance to understand its epidemiology. In this study, we reported the coding-complete genome sequence of BVDV subgenotype 1v strain NX2019/02 in China.

The BVDV non-cytopathogenic strain NX2019/02 was previously isolated from a serum sample of a calf with diarrhea. Whole-blood sample was collected from a calf in Yinchuan city, Ningxia Hui Autonomous Region, in April 2019 and transported on ice to the laboratory. Madin-Darby bovine kidney (MDBK) monolayers in six-well microplates were inoculated with 500 µL of the serum sample and incubated at 37°C for 2 h, replaced with 2 mL DMEM containing 2% horse serum, followed by daily check for 4 days, and blind passaged and examined by indirect immunofluorescence assay (10). Total RNA was extracted from the fifth passage culture using QIAamp Viral RNA Mini Kit (Qiagen, Hilden, Germany). The cDNAs were synthesized by Reverse Transcriptase M-MLV (Takara Biomedical Technology [Beijing] Co., Ltd., Beijing, China) using the primer J2 (Table 1), followed by DNA amplification with ApexHF HS DNA Polymerase FS Master Mix (Dye Plus) (Accurate Biotechnology Co., Ltd., Changsha, China) using the primers for eight overlapping DNA fragments (Table 1). The PCR reaction consisted of pre-denaturation at 94°C for 30 s, 35 cycles at 98°C for 10 s, 55°C for 15 s, and 72°C for 1 min. The PCR products were purified with Zymoclean Gel DNA Recovery Kit (Zymo Research, California, USA) and cloned using pClone007 Versatile Simple Vector Kit (Beijing Tsingke Biotech Co., Ltd) for bi-directional Sanger sequencing on a 3730XL DNA Analyzer. A total of 48 reads were obtained and assembled using the SeqManII software (DNAStar Inc. Madison, WI) for the coding-complete genome sequence of NX2019/02.

**TABLE 1:** Primers used for PCR^*b*^

Primers	Primer sequence (5′−3′)	Positions	Amplicon size (bp)	References
P1F	CAGGTCGACGATTATGCCCTTAG	1–23	2344	This study
P1R	CACAGTATRCCTTGYAACAC	2325–2344		This study
E2F	TGGTGGCCTTATGAGAC	2169–2185	1361	(11)
P7R	CCCATCATCACTATTTCACC	3510–3529		(11)
P2F	ACTTTGAATTTGGACTYTGCC	2655–2685	1836	This study
P2R	TATRACYTTYCTGTGCATRTAGTAC	4466–4490		This study
P3F	TAAGYTGYGTYAGYAGYAAATGG	4405–4427	1710	This study
P3R	GCTATRAATTCYTCTATTGGGTG	6153–6175		This study
P4F	GTTAAGGTAGGRAAGAAYGAAGAG	5598–5621	2176	This study
P4R	GGTAATTCCAAGTYTTRTATGTGTA	7749–7773		This study
P5F	CYCTGGCAACCTACACATAC	7738–7757	2070	This study
P5R	CTACCTCCTTYACWATYCTTG	9787–9807		This study
P6F	GGCTCAAGAARTTCCATRTC	9378–9397	2591	This study
P6R	CATAAGCAGDACYTTCAACC	11950–11969		This study
P7F	GTTATGGGAGTTGGGACGGA	11889–11908	334	This study
J2	ACAGCTAAAGTGCTKWGTGC	12243–12223^*a*^		(12)

^*a*^ The positions of J2 relative to the reference sequence of the NADL strain was described previously (12).

^*b*^ R:(A/G), W:(A/T), D:(A/G/T), and Y:(C/T).

**Fig 1:**
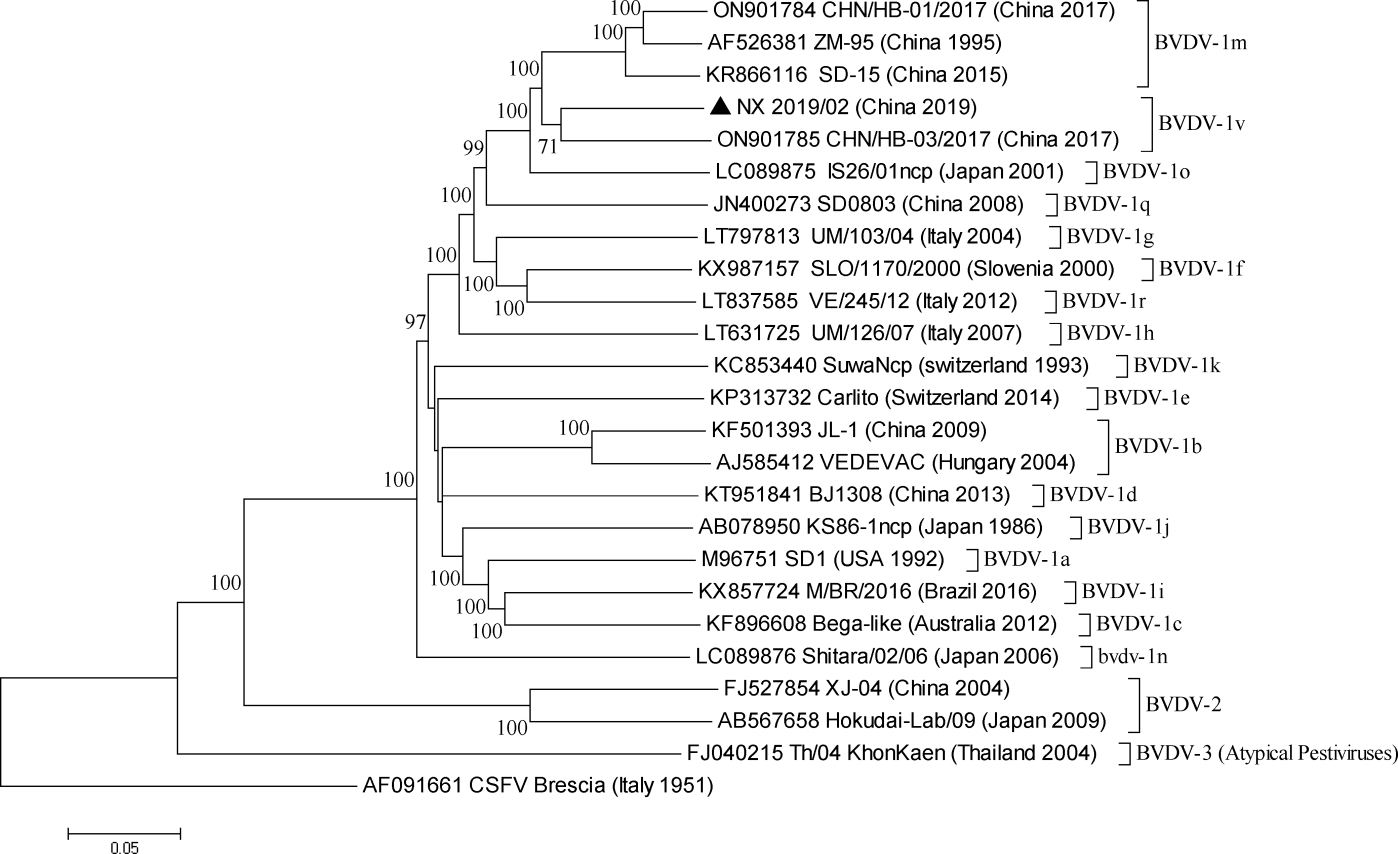
Neighbor-joining phylogeny of BVDV isolates based on coding-complete genome sequences. The classical swine fever virus (CSFV)-rooted phylogenetic tree was constructed by biosoftware MEGA version 11 using the neighbor-joining method and bootstrap analysis (*n* = 1,000). Bootstrap values lower than 70% were removed. The model and parameters were (test: bootstrap method, model: maximum composite likelihood, substitution: transitions plus transversions, uniform rates). Bar indicates nucleotide substitutions per site. The CSFV was used as an outgroup. Selection of the reference sequences was done as described preciously (13). The sequence alignment was performed using the MUSCLE software with default settings (gap open penalty: −400, gap extension penalty: 0).

The polyprotein-coding region of NX20219/02 was 11,700 nt in length and coded 3,900 amino acids. The partially determined 5′UTR and 3′UTR was 290 and 124 nt long, respectively. The NX2019/02 belonged to the 1v clade by phylogenetic analysis (Fig. 1). The 5′UTR of NX20219/02 shared 95.53%–96.33% nucleotide identity with isolates QL1903 (MN849041), GA190608 (MT933204), HN1814 (MN442377), BJ09 (HQ116551), EN-6 (MN417813), and HB-03 (ON901785) within 1v genotype. The structural protein-coding region of NX2019/02 shared a 91.57% nucleotide identity with the HB-03 isolate of the same subgenotype from cattle in the adjacent Hebei province, but their non-structural protein-coding nucleotides were 86.44% identical, resulting in a relatively lower identity (87.98%) at the coding-complete genome level, indicating that there might be a significant undetected genetic diversity of BVDV strains of the subgenotype 1v circulating in China.

## Data Availability

The coding-complete genome sequence of BVDV isolate NX2019/02 has been deposited in GenBank under accession number PQ476186.
